# Risk of conversion to total knee arthroplasty after surgically treated tibial plateau fractures: an observational cohort study of 439 patients

**DOI:** 10.2340/17453674.2024.40605

**Published:** 2024-05-07

**Authors:** Fredrik OLERUD, Anne GARLAND, Nils P HAILER, Olof WOLF

**Affiliations:** 1 Department of Surgical Sciences, Orthopedics, Uppsala University, Uppsala; 2 Department of Orthopedics, Visby Lasarett, Visby, Sweden

## Abstract

**Background and purpose:**

We aimed to assess joint failure rate, i.e., subsequent conversion to TKA after surgical treatment of a tibial plateau fracture (TPF). Secondary aims were to explore the association between joint failure and fracture type, and to determine the risk of failure associated with inadequate joint surface reduction.

**Methods:**

We included all patients ≥ 18 years of age with a surgically treated TPF, treated at Uppsala University Hospital between 2002 and 2015. All fractures were classified according to the Arbeitsgemeinschaft für Osteosynthesefragen/Orthopaedic Traumatology Association (AO/OTA) classification. Postoperative radiographs were evaluated to determine the quality of joint surface inadequate reduction, defined as an articular step-off ≥ 2 mm. The study cohort was linked with the Swedish Arthroplasty Register (SAR) for information on subsequent total knee arthroplasty (TKA).

**Results:**

439 patients (57% women) with a mean age of 55 years (SD 17) were included. According to the AO/OTA classification, the fracture distribution was B1: 4.8%, B2: 10%, B3: 47%, C1: 12%, C2: 6.4%, and C3: 19%. 23 patients (5.2%) were converted to a TKA within 2 years of initial surgery, and 34 patients (7.7%) had been converted by the end of follow-up (16 years). AO/OTA type B3 and C3 had a 6.8 (95% confidence interval [CI] 1.6–29) times greater risk of joint failure compared with B1–2 and C1–C2 at 2 years’ follow-up. Inadequate joint surface reduction led to an 8.4 (CI 3.6–20) times greater risk of conversion to TKA at 2 years’ follow-up.

**Conclusion:**

Overall, 5.2% were converted to a TKA within 2 years. Fracture types AO/OTA B3 and C3 with a comminuted articular surface and inadequate joint surface reduction were strongly associated with joint failure.

The primary goal of surgically treating tibial plateau fractures (TPFs) is to produce a stable joint to allow for early mobilization by addressing surface congruity and axial alignment. TPFs range from a high-energy injury in younger patients to a low-energetic osteoporotic fracture in older adults. In 2000, approximately half of the periarticular fractures of the knee were reported in patients aged ≥ 50 in the population of Edinburgh [1]. These fractures can generally be difficult to reduce and stabilize and are prone to complications, including post-traumatic osteoarthritis (PTOA), non-union, and infection, leading to revision surgery and subsequent conversion to total knee arthroplasty (TKA).

However, patients with TPF vary in age, bone quality, fracture comminution, and degree of displacement. Register-based studies cannot account for particular fracture-related characteristics. A recent single-center study from the UK on TPFs in patients > 60 years with an assessment of radiological fracture features reported a reoperation rate of 12% and an 8% conversion rate to TKA in surgically treated patients during a period of 10 years [2]. A clear association was found between joint depression > 5 mm at the final follow-up and the need for further surgery. No information on the grade of articular comminution was presented. Because certain types of fractures identifiable radiographically in, for example, the hip [3], elbow, and shoulder [4,5] are best treated by joint replacement, studies in which pre- and postoperative radiographs have been examined are warranted. This would help to identify tibial plateau fractures with high risk for failure where another primary treatment may be an option.

We aimed to assess the rate of joint failure, defined as reoperation with TKA, after operative treatment of TPFs, in a single-center cohort. A secondary aim was to explore the association between joint failure, within 2 years after initial surgery, and fracture type with adjustment for age and sex. An additional secondary aim was to assess inadequate joint surface reduction as a risk factor for joint failure.

## Methods

### Study design and variables

Patients ≥ 18 years with a TPF (ICD-10 S82.10 or S82.11) treated surgically at Uppsala University Hospital between 2002 and 2015 with available preoperative radiographs were assessed for inclusion in the study. Exclusion criteria were no Swedish personal identification number (PIN), meaning they would not be registered in the Swedish Arthroplasty Register (SAR), and treatment with primary TKA. In cases with bilateral surgical treatment the second entry was excluded due to statistical technical issues in censoring using survival analysis. Patients who had emigrated or were deceased during the follow-up were noted and censored at the time of expatriation or death.

439 surgically treated patients with TPF with available preoperative radiographs were included in the final cohort of the study. Follow-up ranged from 0 to 16 years, with a mean follow-up of 7.5 years. We chose a 2-year follow-up for the outcome conversion to TKA after TPF because other reports indicate that most conversions related to fractures transpire early in the postoperative period [2,6].

The study cohort was linked to the SAR (formerly known as the Swedish Knee Arthroplasty Register, SKAR) [7] in 2018, resulting in a minimum follow-up of 2 years, based on each individual’s PIN to identify conversion to TKA that could have occurred outside our hospital. The study cohort was described using mean (standard deviation [SD]) for age and sex distribution and categorization into 4 age groups (< 55, 55–64, 65–74, ≥ 75) (Table 1).

**Table 1:** Demographics of the study population of 439 surgically treated patients with a tibial plateau fracture, divided into patients with and without conversion to TKA at 2 years’ follow-up. Values are number of patients and % of the group unless otherwise specified

Characteristics	TKA	No TKA	Total
Number of patients	23	416	439
Mean age (SD)	62.3 (12.9)	54.7 (17.4)	55.2 (17.3)
Age groups			
< 55	6	194 (47)	200 (46)
55–64	7	108 (26)	115 (26)
65–75	6	63 (15)	69 (16)
> 75	4	51 (12)	55 (13)
Female sex	13	236 (57)	249 (57)
Inadequate reduction	13	55 (13)	68 (16)
Schatzker classification			
I	0	12 (2.9)	12 (2.7)
II	10	141 (34)	151 (34)
III	1	39 (9.4)	40 (9.1)
IV	1	30 (7.2)	31 (7.0)
V	5	65 (16)	70 (16)
VI	6	129 (31)	135 (31)
AO/OTA classification			
B1	0	21 (5.0)	21 (4.8)
B2	1	43 (10)	44 (10)
B3	15	193 (46)	208 (47)
C1	1	53 (13)	54 (12)
C2	0	28 (6.7)	28 (6.4)
C3	6	78 (19)	84 (19)

### Fracture classification

Fractures were classified according to the AO/OTA [8] and Schatzker classifications [9] by 1 of the authors (FO). A sample of 171 cases was classified independently by a second observer in order to validate interobserver agreement.

Schatzker classification groups used were I–VI, while the AO/OTA classification localizations, types, and groups were 41 B1-3 and 41 C1-3. No subgroups were used because the cohort would be split into too many groups relative to its size.

### Postoperative reduction

Postoperative radiographs were assessed to determine joint surface reduction and were deemed adequate or inadequate. Inadequate joint surface reduction was defined as a joint surface step-off of ≥ 2 mm on anterior–posterior (AP) or lateral radiographs. Other postoperative measurements, such as alignment, widening of the tibial plateau, and deviant lateral convexity, were not studied. All measurements were conducted on non-calibrated radiographs.

### Outcome measures

This study cohort was linked to the SAR using the unique Swedish PINs to obtain information on conversion to TKA. The SAR has a coverage of 98% and a completeness of 96% for primary TKA performed nationally in Sweden.

### Statistics

Descriptive statistics were used to calculate the proportion of surgically treated TPFs converted to TKA, either within 2 years of initial surgery or at the final follow-up. Kaplan–Meier survival analysis was performed to assess time to event (conversion to TKA as registered in SAR). Criteria for censoring were end of follow-up, death, and expatriation, whichever came first. Causes for censoring were non-informative. The most common cause was end of follow-up. Patients were included between 2002 and 2015 and the linking to SAR was completed in 2018, thus providing a minimum of 2 years’ follow-up.

Cox regression analysis was performed to estimate the association of preoperative fracture classification with the risk of joint failure, with hazard ratios (aHRs) adjusted for age and sex, as both have an impact on bone quality and thus also can be associated with fracture risk and risk of joint failure [1]. We also know that type of trauma such as high- vs. low-energy trauma can impact outcome and type of fracture sustained. Other comorbidities and external factors such as smoking, manifest osteoporosis, and/or treatment with drugs that affect bone quality can be associated with risk of fracture/failure; however, these variables were not present in our material. 95% confidence intervals (CI) were used to describe estimation uncertainty.

To avoid division into inadequately sized subgroups we merged Schatzker I–III (lateral), Schatzker IV–VI (medial and bicondylar), and group only, AO/OTA B and C. A post-hoc analysis, using a fifth merged group using the AO fracture classifications with the most comminuted joint surface (i.e., AO/OTA type B3 + C3, hereafter referred to as “complex articular”), was also evaluated.

Cox regression analysis was also used to estimate the association of postoperative joint surface reduction (adequate and inadequate) with the risk of joint failure adjusted for confounders (i.e., age and sex). Postoperative reduction was studied separately and not included as a confounder in the main analysis, as the study’s goal was to analyze preoperative factors associated with joint failure. Kappa values were calculated to assess interobserver agreement on classification of TPFs. Type-specific kappa was 0.70 for Schatzker and 0.64 for AO. Kappa analysis showed substantial interobserver agreement (i.e., 0.61–0.80) according to categories defined by Landis and Koch [10].

For all Cox regression models Schoenfeld residuals were calculated and plotted to ensure model fit.

A 2-tailed alpha of 0.05 was used in all tests. All statistical analyses were performed using IBM SPSS software (version 27, IBM Corp, Armonk, NY, USA).

### Ethics, funding, data sharing plan, and disclosures

Ethical approval was obtained from the Regional Ethical Committee in Uppsala (Dnr 2017/245), and we complied with the ethical principles of the Helsinki Declaration. The study follows the Strengthening the Reporting of Observational studies in Epidemiology (STROBE) guidelines. No specific grants were received for this study. Data can be made available on reasonable request to the corresponding author, though an approved ethical application is needed as it contains sensitive data from national quality registers. The authors declare no competing interests. Complete disclosure of interest forms according to ICMJE are available on the article page, doi: 10.2340/17453674.2024.40605

## Results

### Characteristics of the study population

**Figure 1:**
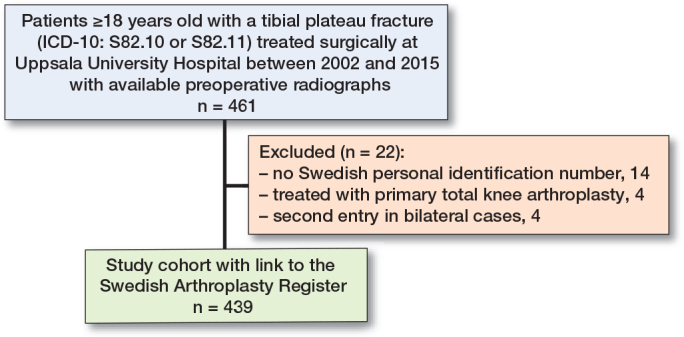
Flowchart of the study population of intra-articular tibial plateau fractures after radiographic review.

Of 461 available patients, 22 were excluded (missing PIN 14, primary TKA 4, second side in bilateral cases 4) leaving a study cohort of 439 patients (Figure 1). 57% of the fractures occurred in women, and the mean age of all patients was 55 years (SD 17). According to the Schatzker classification there were 2.7% type I, 34% type II, 9.1% type III, 7.0% type IV, 16% type V, and 31% type VI fractures. According to the AO/OTA system, there were 4.8% type B1, 10% type B2, 47% type B3, 12% type C1, 6.4% type C2, and 19% type C3 fractures.

14 (3%) of the patients were unable to complete the minimum of 2 years’ follow-up due to unrelated causes. 11 died and 3 emigrated, and were thus lost to follow-up and subsequently censored in survival analyses.

### Conversion to TKA

23 (5.2%) were converted to TKA within 2 years after primary surgery. The conversion rate was 3.0% in patients < 55 years, 6.1% in patients 55–64 years, 8.6% in patients 65–74 years, and 7.1% in patients ≥ 75 years (Table 1). 34 (7.7%) were converted to a TKA during the follow-up period of up to 16 years (mean 7.5 years).

When merging groups, the Schatzker distribution was type I–III in 44% and type IV–VI in 56% of the cases. The AO/OTA distribution was type B in 62% and type C in 38% of the cases.

We found no statistically significant association between fracture type (Schatzker, AO/OTA, merged Schatzker, merged AO/OTA) and the risk of failure for the adjusted (sex, age) models (Table 2, see Appendix).

**Table 2:** Hazard ratio (HR) with 95% confidence interval (CI) for conversion to total knee replacement at 2 years for Schatzker and AO groups of tibial plateau fractures

Group	HR (CI)
Schatzker II	1 reference
Schatzker III	0.35 (0.04–2.8)
Schatzker IV	0.44 (0.1–3.5)
Schatzker V	1.4 (0.5–4.0)
Schatzker VI	0.7 (0.2–1.9)
Schatzker IV–VI (reference Schatzker I–III) ^a^	1.03 (0.4–2.3)
AO B2	1 reference
AO B3	3.5 (0.4–27)
AO C1	0.7 (0.04–12)
AO C3	4.3 (0.5–37)
AO group C1–3 (reference AO B) ^a^	0.72 (0.3–1.7)

Schatzker I and AO B1, C2 had no events.

a Merged Schatzker and AO groups

**Figure 2:**
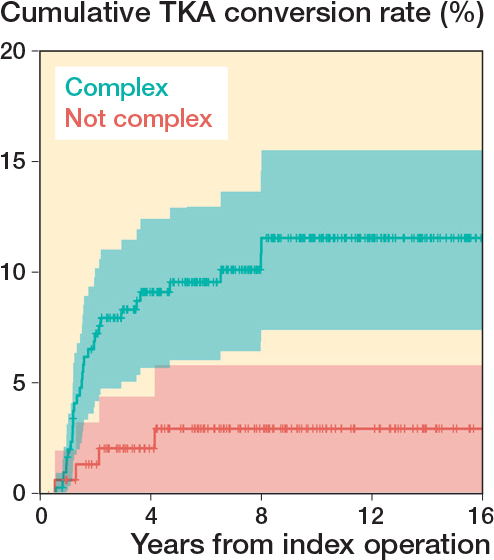
Kaplan–Meier survival analysis of fracture type as a risk factor for joint failure, i.e., conversion to total knee arthroplasty (TKA). Complex fractures are those with a comminuted articular surface (i.e., AO/OTA 41B3 and 41C3 fractures). Highlighted zones represent 95% confidence intervals.

Complex articular fractures (n = 292), comprising AO/OTA 41B3 and 41C3, had a 6.8 times (CI 1.6–29) greater risk of conversion to TKA within 2 years (P = 0.01) and a 4.8 times (CI 1.7–14) greater risk of conversion at the end of follow-up (P = 0.004) when compared with non-complex articular fractures (B1–2 and C1–2) (Table 3, Figure 2).

**Table 3:** Risk of joint failure, i.e., conversion to total knee arthroplasty within 2 years of initial surgery and during the full follow-up

Factor	aHR (CI) ^a^	P value
Inadequate reduction		
2 years	8.4 (3.6–20)	< 0.001
full follow-up	8.6 (4.3–17)	< 0.001
Complex articular fractures ^b^		
2 years	6.8 (1.6–29)	0.01
full follow-up	4.7 (1.7–14)	0.004

a aHR = adjusted Hazard ratios calculated using Cox regression with 95% confidence intervals (CI) and adjusted for age and sex.

b Complex articular fractures = AO/OTA 41B3 and 41C3.

### Joint surface reduction and conversion to TKA

**Figure 3:**
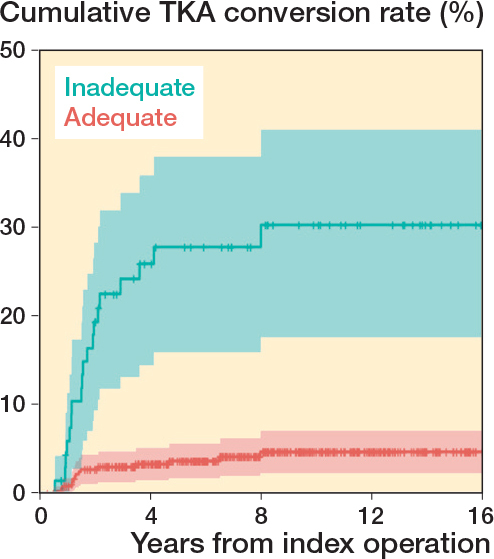
Kaplan–Maier survival analysis of adequate (< 2 mm) vs. inadequate (≥ 2 mm) fracture reduction at first postoperative radiograph after primary TPF surgery. Highlighted zones represent 95% confidence intervals.

69 (16%) patients had an inadequate joint surface reduction. The inadequate joint surface reduction was associated with an 8.4 times greater risk of failure (CI 3.6–20). Patients with inadequate joint surface reduction had an approximate rate of conversion of 30% (Table 3, Figure 3).

## Discussion

We aimed to assess the rate of conversion to TKA after TPF and to gain a deeper understanding as to which fracture characteristics are associated with joint failure in operatively treated TPFs.

We found a TKA conversion rate of 5.2% within 2 years after primary surgery and a conversion rate of 7.7% at follow-up up to 16 years. Complex articular fractures, i.e., fractures with comminution, were a risk factor for joint failure in our population. Additionally, inadequate joint surface reduction was associated with an 8.4 times greater risk of subsequent conversion to TKA.

Our results of a TKA conversion rate of 5.2% within 2 years of initial treatment are in line with previous studies [11,12]. Numerous reports have investigated the presence of radiographic PTOA after TPFs, with proportions ranging from 17% to 73% [13-19]. For bicondylar fractures, even higher rates have been reported [13-19]. The incidence of PTOA may rise in correlation with increasing Schatzker classification [18]. However, we believe that the conversion rate to TKA is a better measurement than radiographic OA. The SAR, with an approximate completeness of 96%, is considered a reliable source of information on conversion. All knee arthroplasties in Sweden are registered, regardless of underlying diagnosis [7].

3 major register-based studies concerning TKA conversion rates in TPFs have been conducted. Elsoe et al. studied 7,950 TPFs, regardless of treatment, from the Danish Patient Register and found a 5.7% conversion rate to TKA with a mean follow-up of 13.9 years [11]. A Finnish hospital discharge register report found a conversion rate to TKA of 4.4% in 7,701 patients after TPF with a mean follow-up of 5.1 years [20]. The conversion rate was 5.0% in the surgically treated group. In comparison with these studies, our conversion rate of 5.2% at 2 years follow-up and 7.7% at full follow-up is on the good side, given that the study cohort also includes regional referrals for operative treatment at our University Hospital, in addition to patients from the primary trauma uptake area. Consistent with previous findings, we found a modest conversion rate. Another single-center study of 220 patients ≥ 60 years with TPFs, of whom 40% were treated operatively, reported a total conversion rate to TKA of 8% [2]. The mean joint depression in the operatively treated patients was 3.2 mm at final follow-up, and 10 of 86 surgically treated patients were converted to TKA (12%) at a mean of 10 months after injury. This conversion rate is somewhat higher than in our cohort, even when looking only at patients aged 65–74 years with a conversion rate of > 8%. In our cohort the TKA conversion rate decreased in patients ≥ 75 years, suggesting that this patient group may have lower demands and does not wish to undergo further operative procedures. However, due to the risks associated with TKA procedures, in some patients with joint failure subsequent TKA is still not recommended. This may for instance be the case in patients with multiple comorbidities in whom the risk of postoperative complications is severely increased. A gap or step off in the joint surface > 2 mm may still lead to a good functional outcome. Vaartjes et al. recently showed no difference in functional outcome in conservatively treated, minimally displaced TPFs with a step-off between 2 and 4 mm [21].

Wasserstein et al. studied 8,426 cases of surgically treated TPFs with a 10-year follow-up using billing codes in Ontario, Canada [12]. They found a conversion rate of 7.3% after 10 years. The conversion rate was only 0.32% within 2 years after primary fracture surgery. Factors influencing conversion rate can be national or regional algorithms on primary treatment, access to physiotherapy, national or regional algorithms on the timing of conversion, or reoperation after unsuccessful primary treatment.

The merged group “complex articular fractures” was associated with a higher risk of joint failure. This group was created because of the small number of conversions and the radiographic similarities between these 2 types with comminuted articular surfaces. One hypothesis of this study was that the more comminution of the fracture, the greater the risk of joint failure. AO/OTA type C1 includes only a simple articular component and can have an undisplaced metaphyseal fissure, making it an uncomplicated fracture in the younger patient with healthy bone. A B3 fracture, on the other hand, can include a completely crushed articular surface with substantial depression of the joint surface and major bone loss, making it a serious surgical challenge.

The most important factor in the surgical treatment of TPFs is to produce a stable fixation and a congruent joint. However, quantifying its importance is lacking in the literature. In our material we found a significantly increased risk of subsequent failure, i.e., conversion to TKA, with an articular step-off ≥ 2 mm, as measured on the first postoperative radiograph. This limit was chosen as it was deemed enough to be easily measured on plain radiographs and has been reported, together with a mechanical axis of > 4° varus, to be a strong predictor of inferior outcomes after medial TPFs [22]. Singleton et al. showed that a residual articular depression of < 2.5 mm resulted in significantly smaller losses in knee range of motion and better Oxford, Iowa, and KOOS scores, while no significant difference in the above-mentioned parameters was shown dependent on mechanical axis alignment [23]. Barei et al. investigated 41 bicondylar fractures and found an association between a satisfactory joint surface reduction (< 5 mm) and improved outcome scores [24].

### Strengths and limitations

Our study has a large cohort size (n = 439) of surgically treated patients, although the low conversion rate made the association of fracture type with the risk of failure uncertain. We assessed fracture type and postoperative reduction on individual radiographs. Moreover, the linking to the SAR resulted in the best possible control of the conversion to TKA, which can always be performed at another healthcare institution without insight from the primary treatment center. This study is the largest single-center study conducted with radiographic analysis. The SAR, with its high coverage and completeness, results in a more complete follow-up than in many other cohort studies, where uncertainty regarding the endpoint “conversion to TKA” remained [2,12]. Other studies using registers have reported on larger cohorts of patients with TPFs [11,20]. However, individual fracture characteristics were not recorded in either of the studies. In one of these studies initial treatment was not recorded and patients were followed for up to 20 years, which would make idiopathic OA a possible cause of TKA [11]. Laterality was not obtainable in either of the studies, although one of the studies did adjust for laterality in their data [20]. No radiographs were analyzed in either of these studies. Wasserstein et al. identified 8,426 cases of surgically treated TPFs, making their cohort substantially larger than ours [12]. However, no preoperative fracture characteristics were included in the study. It was not possible to verify laterality of potential arthroplasty and no radiographic analysis was performed. Only patients subsequentially treated in the Ontario area were identified.

The injury mechanism was not studied. All patients ≥ 18 years were included, with 200 (46%) of 439 patients being < 55 years of age at the time of injury. In such a group of young patients TKA is the last resort of treatment, and thus the 2-year follow-up may be too short. A comminuted fracture resulting from low energetic trauma in the osteoporotic patient has a higher risk of failure than the younger patient with bone that is more amenable to screw fixation. Regrettably, the limited material made it impossible to draw valid conclusions had elderly patients been analyzed separately.

Other possible confounders that could not be accounted for were presence of manifest osteoporosis at time of injury and the patients’ smoking status. Both osteoporosis and smoking can be associated with fracture type and risk of failure.

Classifications and measurements of reduction adequacy were all made on plain radiographs. This was done because of the reproducibility and consistency. Classifications were originally validated on plain AP radiographs, and not all patients had preoperative CT scans. However, in clinical practice nowadays, the gold standard is to assess preoperative CT scans as there are numerous three-dimensional factors that influence preoperative planning [25].

Primary TKA for TPFs is considered a valid treatment option. However, the consensus is that this should be done cautiously and only after individual consideration. Severely displaced and comminuted fractures tend to accompany severe metaphyseal destruction and cortical fissures, making endoprosthesis weight-bearing difficult. These fractures often necessitate the use of revision or tumor prostheses and thus limit this option to centers with sufficient competence and resources [26].

### Conclusion

We found a conversion rate to TKA was 5.2% within 2 years after primary surgery for TPF. In our material inadequate joint surface reduction and increased comminution seems to be associated with a higher risk of conversion to TKA. These subgroups warrant further studies as to how they are best treated.

## Notes

FO, AG, NPH, and OW planned the study. FO wrote the initial draft and performed the analyses. All authors contributed to the interpretation of the data and revision of the manuscript.

Handling co-editors: Marianne Westberg and Philippe Wagner

Acta thanks Gunnar Birkeland Flugsrud and Morten Schultz Larsen for help with peer review of this manuscript.
